# Localization and Discrimination of the Perturbation Signals in Fiber Distributed Acoustic Sensing Systems Using Spatial Average Kurtosis

**DOI:** 10.3390/s18092839

**Published:** 2018-08-28

**Authors:** Fei Jiang, Honglang Li, Zhenhai Zhang, Yixin Zhang, Xuping Zhang

**Affiliations:** 1 School of Mechatronics Engineering, Beijing Institute of Technology, Beijing 100081, China; flyjiang92@gmail.com; 2 Institute of Acoustics, Chinese Academy of Sciences, Beijing 100190, China; 3 The Key Laboratory of Intelligent Optical Sensing and Manipulation, Nanjing University, Nanjing 210008, China; zyixin@nju.edu.cn (Y.Z.); xpzhang@nju.edu.cn (X.Z.)

**Keywords:** fiber distributed acoustic sensing, Φ-OTDR, localization, discrimination, kurtosis

## Abstract

Location error and false alarm are noticeable problems in fiber distributed acoustic sensing systems based on phase-sensitive optical time-domain reflectometry (Φ-OTDR). A novel method based on signal kurtosis is proposed to locate and discriminate perturbations in Φ-OTDR systems. The spatial kurtosis (SK) along the fiber is firstly obtained by calculating the kurtosis of acoustic signals at each position of the fiber in a short time period. After the moving average on the spatial dimension, the spatial average kurtosis (SAK) is then obtained, whose peak can accurately locate the center of the vibration segment. By comparing the SAK value with a certain threshold, we may to some degree discriminate the instantaneous destructive perturbations from the system noise and certain ambient environmental interferences. The experimental results show that, comparing with the average of the previous localization methods, the SAK method improves the pencil-break and digging locating signal-to-noise ratio (SNR) by 16.6 dB and 17.3 dB, respectively; and decreases the location standard deviation by 7.3 m and 9.1 m, respectively. For the instantaneous destructive perturbation (pencil-break and digging) detection, the false alarm rate can be as low as 1.02%, while the detection probability is maintained as high as 95.57%. In addition, the time consumption of the SAK method is adequate for a real-time Φ-OTDR system.

## 1. Introduction

Fiber distributed acoustic sensing systems based on phase-sensitive optical time domain reflectometry (Φ-OTDR) have been widely used in many fields such as oil and gas pipeline monitoring, structure health monitoring, and perimeter security [1,2,3,4] due to their high sensitivity, large dynamic range, fully distributed manner, and simple configuration. The principal of Φ-OTDR is based on the interference of Rayleigh scattering lights returned from the fiber. The phase of the Rayleigh scattering lights can be affected by external vibration events, which will result in the amplitude variation of the Rayleigh scattering traces. In Φ-OTDR systems, locating the vibration events is an essential problem. Ideally, the vibration location can be obtained by subtracting a Rayleigh trace from an earlier stored trace [1]. However, this method may cause false locations due to the amplitude fluctuation in Rayleigh traces and the system noise of Φ-OTDR.

Various signal processing methods have been introduced to solve this problem. Some focus on the time-domain signal denoising. For example, Lu et al. proposed the moving averaging and moving differential method to reduce the noise power and also increase the frequency response range of the system [5]. Qin et al. introduced a wavelet denoising method to reject the random noises induced by varied polarization states in different position and detectors [6]. Shi et al. [7] and Qin et al. [8] proposed using empirical mode decomposition (EMD) to improve the signal-to-noise ratio (SNR) of location information for the vibration events. Qin et al. also proposed a curvelet denoising method to reduce the time domain noise and improve the detection performance of Φ-OTDR systems [9]. Ölçer et al. presented an adaptive temporal matched filtering method to reduce the effect of fading noise without any impact on the frequency response of the detection system [10]. Some locate the perturbations using the 2-D image characters of Rayleigh traces. For instance, two-dimensional edge detection can improve both the spatial resolution and the SNR of location information [11,12]. Adaptive 2-D image processing method of bilateral filtering algorithm can also enhance the SNR of vibration/intrusion location in Ф-OTDR [13]. Some pay attention to the spatial frequency information of the Rayleigh signals while locating, such as [14,15,16], which can simultaneously extract the vibration location and frequency information with an improved SNR. In addition, correlation dimension method has been proposed to solve the coherent fading problem and improve the multiple location results’ standard deviation [17].

However, the methods above are sensitive to all kinds of perturbations. Perturbations caused by environmental interference may be located as well. For instance, the fiber cables buried underground may be influenced by pedestrians, vehicles, and trains; the fiber cables hanging in the air are usually perturbed by the wind and rain; and fibers that are embedded in concrete and stuck to pipelines could be affected by natural vibrations of the structures. All of these harmless perturbations may also cause the same disturbance results, which may obscure the real harmful perturbations and cause high false alarm rates (FARs). A new method based on multi-scale wavelet decomposition and backpropagation neural network [18] has been proposed to solve this problem. It can locate the perturbations by multi-scale wavelet decomposition and determine them by a 3-layer BP neural network. The results show that the FAR is decreased efficiently. In addition to this method, various event recognition methods [19,20,21,22] have been proposed to discriminate the signals in Φ-OTDR systems.

In this paper, a new localization and discrimination method based on the spatial kurtosis of Rayleigh signals is introduced for Φ-OTDR systems. The spatial kurtosis (SK) along the fiber is first obtained by calculating the kurtosis at each position of the fiber in a short time period, and then spatial average kurtosis (SAK) can be obtained by computing the moving average of SK on the spatial dimension. As a result, both the SK peaks and the SAK peaks may show the perturbation locations. Comparing with SK method, the SAK method can reduce the location error caused by coherent fading problem. We can also simply evaluate whether the perturbation is instantaneous destructive by comparing the SAK value with a certain threshold to some degree. This is because instantaneous destructive perturbations generally have high positive kurtosis, while stationary environmental noises usually have low negative kurtosis. Experimental results show that, the SAK method has higher SNR and lower location standard deviation compared with the previous localization methods. The time consumption of SAK method is low enough to achieve perturbation localization and discrimination in real-time, which is promising for a practical Φ-OTDR system.

## 2. Methods

### 2.1. Kurtosis of Acoustic Signals in Phase-Sensitive Optical Time-Domain Reflectometry

**Figure 1:**
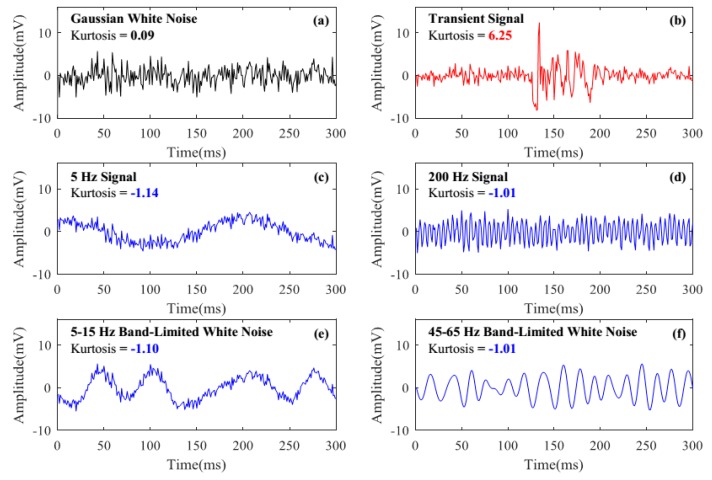
The simulated signals in phase-sensitive optical time-domain reflectometry (Φ-OTDR) systems. (**a**) Gaussian noise; (**b**) Transient signal; (**c**) Signal with the frequency of 5 Hz; (**d**) Signal with the frequency of 200 Hz; (**e**) 5–15 Hz band-limited white noise; (**f**) 45–65 Hz band-limited white noise.

The kurtosis is a fourth-order statistic that can be used to describe the shape of a probability distribution of a random variable. It is defined as:

(1) $$Kurt\left[X\right]=\frac{E\left[{\left(X-\mu \right)}^{4}\right]}{{\left(E\left[{\left(X-\mu \right)}^{2}\right]\right)}^{2}}-3,$$

where *μ* is the mean of random variable *X*, and *E* [*t*] represents the expected value of quantity *t*. The kurtosis of any univariate normal distribution is 0. Thus we can evaluate a distribution by comparing its kurtosis with 0. Distributions with a positive kurtosis (called leptokurtic distributions) have infrequent extreme deviations (or outliers), while distributions with a negative kurtosis (called platykurtic distribution) produce no outliers. In order to analyze the kurtosis of Φ-OTDR signals, we simulated these three main types of signals in Φ-OTDR systems:

(1) Random noise. In Φ-OTDR systems, noises are mainly caused by phase noise of the laser, partial interferometric problem, and electrical noises such as thermal noise and shot noise [5]. These noises nearly follow a normal distribution, thus their kurtosis should be around 0. A Gaussian white noise with the amplitude of 2 mV is used to simulate the random noise in Φ-OTDR systems, shown in Figure 1a. Its kurtosis is 0.09.
(2) Instantaneous destructive perturbations. These signals (structural cracks, gas explodes, excavation damage, destructive intrusions, etc.) are virtually short-time and non-stationary, whose frequency distribution change rapidly over time. They generally have certain extreme values, thus their kurtosis should be positive. Transient signal in Figure 1b is used to simulate the instantaneous destructive perturbations, which is generated by a 5–400 Hz broadband damped sinusoidal signal plus a Gaussian white noise with the amplitude of 1 mV, and the kurtosis of it is 6.25.
(3) Environmental interferences. These signals (wind, the vehicle engine, structural free vibration, etc.) are mostly continuous and stationary, whose frequency distribution is fixed in a short time period. They usually have no extreme values, thus their kurtosis should be negative. Signals in Figure 1c–f are used to simulate environmental interferences. Signals in Figure 1c,d are simulated by a sinusoidal signal with the amplitude of 1 mV plus a Gaussian white noise with the amplitude of 1 mV, which are used to simulate the natural vibrations of the structures. Their sinusoidal signal frequencies are 5 Hz and 200 Hz and their kurtosis are −1.14 and −1.01 respectively. Signals in Figure 1e,f are band-limited white noises, which are used to simulate the environmental interferences such as wind. Their frequency band are 5–15 Hz and 45–65 Hz and their kurtosis are −1.10 and −1.01, respectively.

We can see that the kurtosis of random noise is around 0, and the reason why it is not totally equal to 0 is that the length of it is too short (only 300). We can also see that signals caused by certain stationary environmental interferences have negative kurtosis, while transient signal (corresponding to instantaneous destructive perturbations) has high positive kurtosis. Thus we may discriminate such types of signals in Φ-OTDR systems by comparing their kurtosis with zero.

### 2.2. Spatial Kurtosis of Rayleigh Optical Time-Domain Reflectometry Traces

In Φ-OTDR systems, the Rayleigh backscattering signals can be composed into a matrix. The data picked up from each Rayleigh backscattering trace with the same time delay will form the time series of the corresponding sensing point. Suppose that each Rayleigh backscattering trace has *L* sampling points, which are determined by the sampling rate of the data acquisition card and the fiber length, the matrix with *N* received Rayleigh backscattering traces can be defined as:

(2) $$R_{N\times L}=\left[\begin{array}{cccc} x_{1}^{1} & x_{2}^{1} & \cdots & x_{L}^{1}\\ x_{1}^{2} & x_{2}^{2} & \cdots & x_{L}^{2}\\ \vdots & \vdots & \ddots & \vdots \\ x_{1}^{N} & x_{2}^{N} & \cdots & x_{L}^{N} \end{array}\right],$$

where $x_{l}^{n}$ represents the amplitude of Rayleigh signal at position *l* at time *n*.

We accumulated *w* adjacent Rayleigh traces as one processing unit to calculate the kurtosis of the amplitude signal. The kurtosis of the signal at position *l* at time *n* can be defined as:

(3) $$k_{l}^{n}=\frac{\frac{1}{w}\sum_{i=n-w+1}^{n}{\left(x_{l}^{i}-\frac{1}{M}\sum_{i=n-w+1}^{n}x_{l}^{i}\right)}^{4}}{{\left(\frac{1}{w}\sum_{i=n-w+1}^{n}{\left(x_{l}^{i}-\frac{1}{w}\sum_{i=n-w+1}^{n}x_{l}^{i}\right)}^{2}\right)}^{2}}-3,$$

where *w* can be also regarded as the window size of kurtosis computing, which determines the temporal resolution of the localization and discrimination. The spatial kurtosis of the Rayleigh traces at time *n* is defined as:

(4) $$SK\left(n\right)=\left[k_{1}^{n},k_{2}^{n},\cdots ,k_{L}^{n}\right].$$

By identifying the local maxima and minimum of SK at time *n*, we could estimate whether there are perturbations and locate them accurately on the sensing fiber. Moreover, we introduced a time step *s* to reduce the computation in real-time Φ-OTDR systems. It means that the time interval of calculating SK is *s* × *fs* (where *fs* represents the pulse repeat frequency). Composing the SK vectors at different times into a new matrix

(5) $$SK=\left[\begin{array}{cccc} k_{1}^{1} & k_{2}^{1} & \cdots & k_{L}^{1}\\ k_{1}^{1+s} & k_{2}^{1+s} & \cdots & k_{L}^{1+s}\\ k_{1}^{1+2s} & k_{2}^{1+2s} & \cdots & k_{L}^{1+2s}\\ \vdots & \vdots & \cdots & \vdots \end{array}\right],$$

a two-dimensional image of SK over the monitoring duration can be obtained. Then the perturbation locations could be identified clearly from this image.

### 2.3. Moving Average on the Spatial Dimension

**Figure 2:**
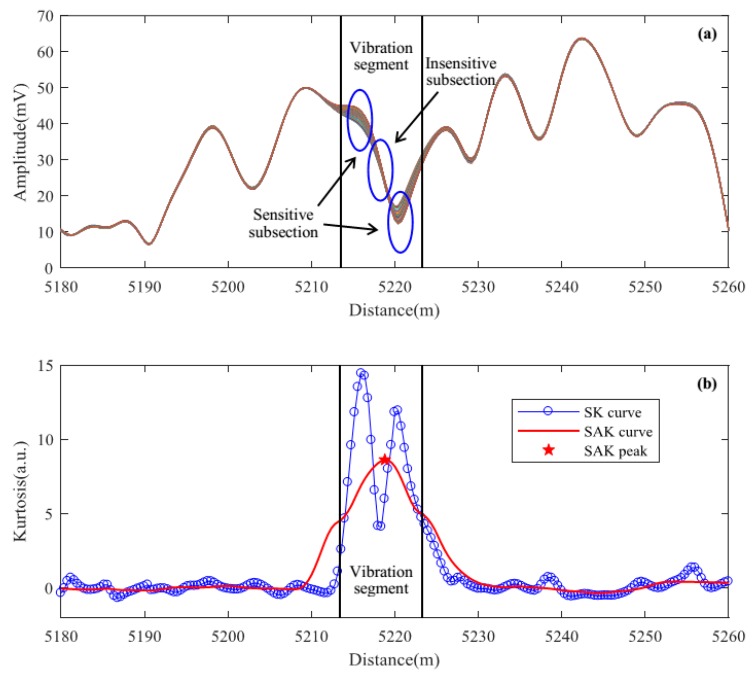
The vibration locating results when coherent fading problem exists. (**a**) 100 averaged Rayleigh traces; (**b**) The spatial kurtosis (SK) and special average kurtosis (SAK) curves of the Rayleigh traces.

In Φ-OTDR systems, the accurate location of perturbations can be figured out by [7]:

(6) $$z=z_{cent}-\frac{T_{p}v}{4},$$

where $z_{cent}$ is the center of the vibration response segment, $T_{p}$ is the optical pulse width, and *v* is the light velocity in fiber. In order to obtain the accurate location of the perturbation in Φ-OTDR systems, we should figure out the center of the vibration segment. However, some subsections of the fiber vibration segment may be in insensitive states due to the coherent fading problem [23]. Figure 2a shows 100 consecutive averaged Rayleigh traces, which were acquired by our Φ-OTDR system. The optical pulse width used here is 100 ns, which means that the spatial resolution is 10 m. The vibration segment is from 5213 m to 5223 m. We can see that two subsections of the vibration segment are in sensitive state while the middle subsection is in insensitive state. This may make it difficult to locate the accurate vibration segment center and result in a location error and a bad locating repeatability.

For the SK localization method this problem exists as well. As shown in Figure 2b, the SK curve of the original Rayleigh traces splits into two peaks in the vibration segment. The two SK peaks correspond to the two sensitive subsections in Figure 2a, while the SK valley corresponds to the insensitive subsection in Figure 2a. It is hard to figure out the accurate vibration location by the SK curve. Virtually, we can solve this problem by calculating the moving average of SK on the spatial dimension. The result can be called SAK, which is defined as:

(7) $$SAK\left(n\right)=\frac{1}{M}\left[\sum_{l=1}^{M}k_{l}^{n},\sum_{l=2}^{M+1}k_{l}^{n},\cdots ,\sum_{l=L-M+1}^{L}k_{l}^{n}\right]$$

where *M* represents the moving average number, which depends on $T_{p}$ and the data acquisition card (DAQ) sampling rate (SR) of the Φ-OTDR system. It should be equal to the number of sampling points in an optical pulse width ($M=T_{p}\times SR$). When the vibration occurs, the location of SAK peak should be the center of the vibration segment. We can see from Figure 2b that the SAK peak virtually locates at the vibration segment center. Then we can obtain the accurate location of the vibration according to Equation (6).

## 3. Experimental Results

### 3.1. Experimental Setup

**Figure 3:**
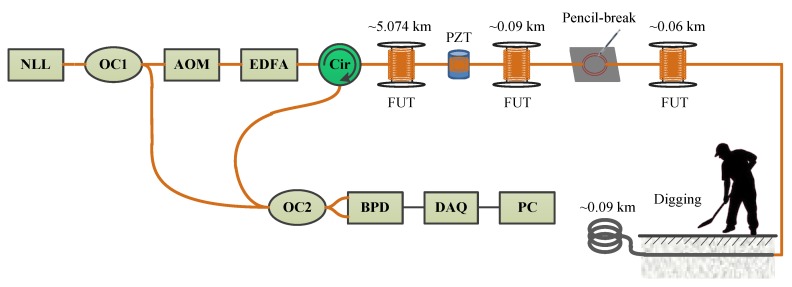
The experimental setup. NLL: narrow line-width laser; OC: optical coupler; AOM: acoustic-optic modulator; EDFA: Erbium-doped fiber amplifier; Cir: circulator; FUT: fiber under test; BPD: balanced photo detector; DAQ: data acquisition; PC: personal computer.

The experimental setup of our Φ-OTDR system is shown in Figure 3. A narrow line-width laser (NLL) is used as the light source. The laser light is divided into two parts by a 10:90 optical coupler (OC1). Then 10% of the light is used as the local light to perform a coherent detection, and 90% of the light is modulated into optical pulses with a 200 MHz frequency shift by an acoustic optical modulator (AOM). After being amplified by an erbium-doped fiber amplifier (EDFA), the optical pulses are launched into an optical circulator. The Rayleigh backscattering light from the sensing fiber then interferes with the local light at a 50:50 optical coupler (OC2) and result in a beat light. The beat light is then detected by a balanced photodetector (BPD) through a differential way, which could improve the SNR by 3 dB [4]. The output electric signal from the BPD is then sampled by a DAQ with a 1 GS/s sampling rate. The Rayleigh trace amplitude is finally demodulated by Hilbert transform and downsampled to 250 MS/s to reduce the computation load. In our experiments, the pulse repeat frequency is 1 kHz and the pulse width is 100 ns, which means that the maximum frequency response and the spatial resolution of our system are 500 Hz and 10 m respectively.

Both indoor experiment and outdoor experiment were conducted to evaluate the proposed method. In the indoor experiment, a piezoelectric transducer (PZT) cylinder glued with 10 m long fiber was added after the ~5.074 km long fiber under test (FUT) in our system. The PZT was used to simulate the environmental interferences such as structural free vibration and vehicle engine. Pencil-break vibration was used to simulate the civil structure crack, which was felt by a 2 m long fiber loops glued to a flame retardant (FR-4) plate. The pencil-break vibration was conducted by an HB pencil lead with 0.5 mm diameter. The fiber loops was put at ~5.164 km. In the outdoor experiment, a ~100 m long fiber cable was placed outdoor. The first ~10 m of it was buried beneath a flower bed with 0.3 m deep, while the rest ~90 m of it was placed in a corner. Digging the soil near the buried fiber cable was inducted as the instantaneous destructive perturbations. The flower bed is near to the road, so the fiber cable buried in the soil might be affected by the pedestrians and vehicles, which could be regard as the harmless perturbations.

### 3.2. Localization Results of Instantaneous Destructive Perturbations

A total of 200 tests, including 100 pencil-break tests (50 of which were under 100 ns pulse width and 50 of which were under 500 ns pulse width) and 100 digging tests (50 of which were under 100 ns pulse width and 50 of which were under 500 ns pulse width) were carried out to evaluate the localization performance of proposed method. The pencil-break tests and digging tests were performed at two fixed positions (~5164 m for pencil-break and ~5220 m for digging).

In this part, we compared the proposed method with four previous methods, including moving average and differential (MAD) [5], two-dimensional edge detection (TED) [11], wavelet denoising (WD) [6], and multi-scale wavelet decomposition (MWD) [18]. The comparisons include locating SNR, locating repeatability, and time consumption. The parameters of these methods are tuned to get the relatively high locating SNR. The moving average number of MAD was 30; the Sobel operator size of TED was 3 × 3 for pencil-break and 5 × 5 for digging; the wavelet basis function of WD and MWD was db3, and the decomposition level was 6. The window length used to compute kurtosis was 300, the time step was 150, and the moving average number of SAK was 25.

#### 3.2.1. Signal-to-Noise Ratio Improvement of the Locating Information

**Figure 4:**
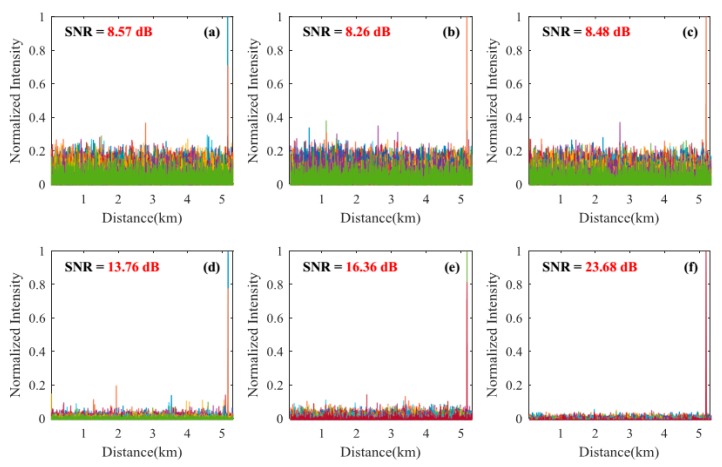
The locating results of pencil-break vibration under 100 ns pulse width. (**a**) The result of moving average and differential (MAD) method; (**b**) The result of two-dimensional edge detection (TED) method; (**c**) The result of multi-scale wavelet decomposition (MWD) method; (**d**) The result of wavelet denoising (WD) method; (**e**) The result of SK method; (**f**) The result of SAK method. SNR: signal-to-noise ratio.

**Figure 5:**
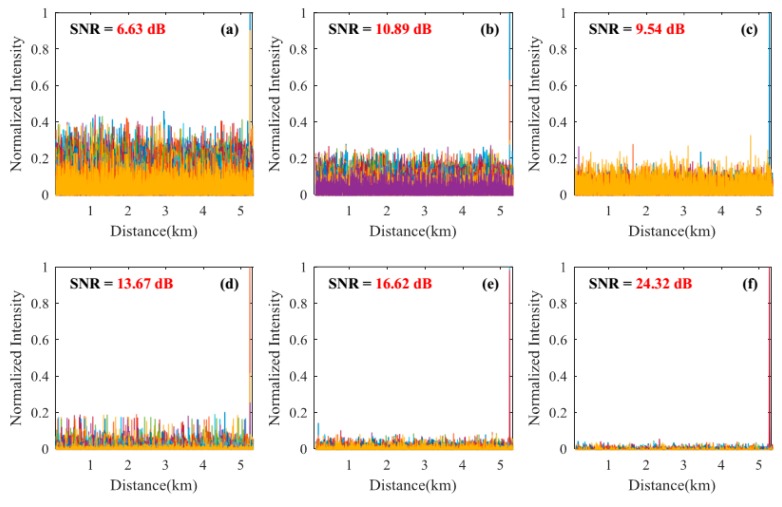
The locating results of digging event under 100 ns pulse width. (**a**) The result of MAD method; (**b**) The result of TED method; (**c**) The result of MWD method; (**d**) The result of WD method; (**e**) The result of SK method; (**f**) The result of SAK method.

**Figure 6:**
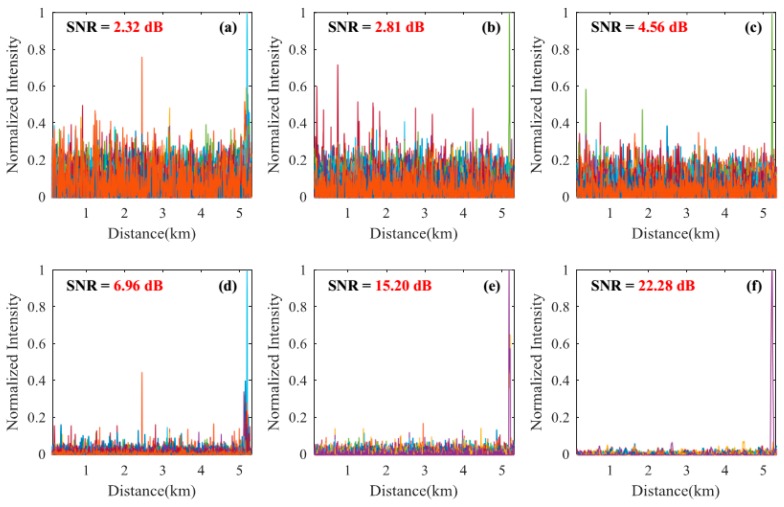
The locating results of pencil-break under 500 ns pulse width. (**a**) The result of MAD method; (**b**) The result of TED method; (**c**) The result of MWD method; (**d**) The result of WD method; (**e**) The result of SK method; (**f**) The result of SAK method.

**Figure 7:**
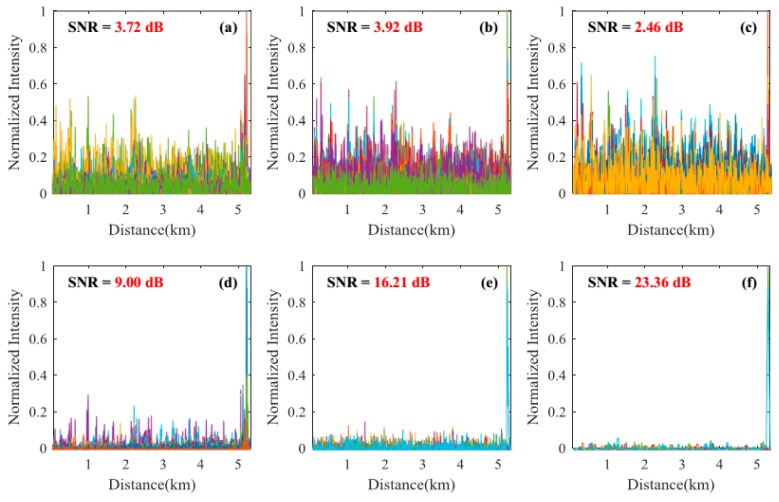
The locating results of digging event under 500 ns pulse width. (**a**) The result of MAD method; (**b**) The result of TED method; (**c**) The result of MWD method; (**d**) The result of WD method; (**e**) The result of SK method; (**f**) The result of SAK method.

Locating results of pencil-break vibration and digging are shown in Figure 4, Figure 5, Figure 6 and Figure 7. Here, SNR is defined as the ratio between the signal peak intensity (S) and the maximum background noise intensity (N) with the equation of 20 lg(S/N) [18]. Figure 4 and Figure 5 respectively show the locating results of pencil-break vibration and digging under 100 ns pulse width. We can see that the SAK method obtains the highest SNR (23.68 dB for pencil-break and 24.32 dB for digging) among all the methods. Figure 6 and Figure 7 respectively show the locating results of pencil-break vibration and digging under 500 ns pulse width. Likewise, the SAK method has the highest SNR (22.28 dB for pencil-break and 23.36 dB for digging) among all the methods. We can see that when the pulse width is increased from 100 ns to 500 ns, the SNR of MAD, TED, and MWD significantly reduce, whereas the SNR of SK and SAK maintain high. The SNR of SAK method is higher than SK, which means that moving average on the spatial dimension may improve the locating SNR.

The average locating SNRs of each method on the 200 locating tests are shown in Table 1. We can see that, comparing with the average of the MAD, TED, WD, and MWD methods, the SAK method improves the pencil-break locating SNR by 13.95 dB (under 100 ns pulse width) and 16.6 dB (under 500 ns pulse width); and the digging locating SNR by 10.60 dB (under 100 ns pulse width) and 17.3 dB (under 500 ns pulse width).

#### 3.2.2. Locating Accuracy

**Figure 8:**
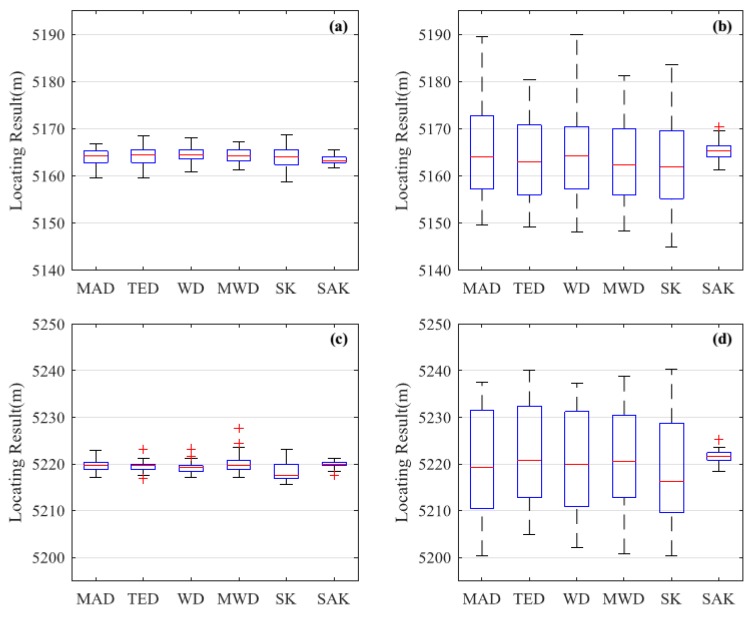
The statistic results of locating results. (**a**) The result of pencil-break vibration under 100 ns pulse width; (**b**) The result of pencil-break vibration under 500 ns pulse width; (**c**) The result of digging under 100 ns pulse width; (**d**) The result of digging under 500 ns pulse width.

The box plot of locating results is shown in Figure 8. We can see that for both pencil-break and digging, the range of the locating results gets wider when the pulse width increases from 100 ns to 500 ns. Thus the increasing pulse width may degrade the locating accuracy. We can also see that the SAK method has more consistent results than other methods for both pencil-break vibrations locating and digging events locating. Quantitatively, the averages and standard deviations of pencil-break and digging locating results by the 6 methods under two different pulse widths are shown in Table 2. We can see that the averages of the locating results by these methods show no significant differences, which is because for all of the methods the locating results may fluctuate above and below the identical real locations. However, the standard deviations of the locating results differ. We can see that the SAK method has the lowest standard deviation for both pencil-break and digging locating. When the pulse width is 100 ns, the standard deviation of the pencil-break and digging locating results by SAK method reduce from 1.7 m and 1.4 m (the average locating standard deviation of the previous methods) to 0.8 m and 0.7 m, respectively. Two-sample F-tests show that this improvement is statistically significant for all of the previous methods (p<0.0056). When the pulse width is 500 ns, the pencil-break and digging locating standard deviation of SAK method are only 1.9 m and 1.8 m respectively, while the average locating standard deviation of the previous methods are as high as 9.2 m and 10.9 m respectively. Two-sample F-tests show that this improvement is also statistically significant for all of the previous methods ($p<10^{-19}$). Comparing with SK method, the SAK method decreases the localization standard deviation of pencil-break and digging under 500 ns pulse width by 8.4 m and 10.4 m, respectively, which means that moving average on the spatial dimension can effectively improve the locating accuracy.

#### 3.2.3. Time Consumption

**Figure 9:**
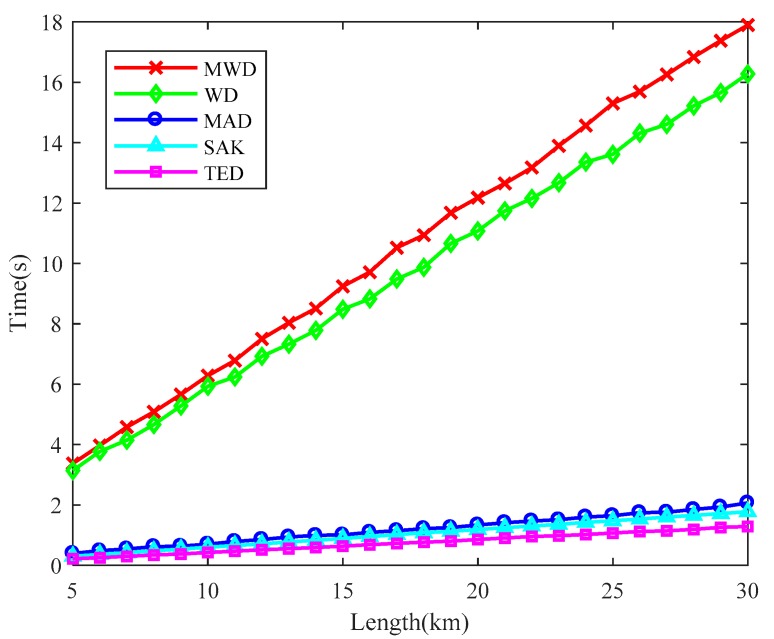
The time consumption of various locating methods under different fiber length.

For a real-time Φ-OTDR system, the locating speed should be faster than the data generating speed. Therefore, the time consumption of the localization algorithm should be as less as possible. We compared the time cost of SAK method with the previous locating methods under different fiber length, shown in Figure 9. The methods are performed under 5000 Rayleigh traces (corresponding to 5 s long data in our system). The CPU used in our PC is an Intel Core i7-4930K. The algorithms are executed by MATLAB R2017a (MathWorks, Inc., Natick, MA, USA). We can see that the time consumption of SAK method is close to that of the MAD and TED methods and it outperforms both the WD and MWD methods. When the fiber length is 30 km (the spatial sampling interval is 1 m), the SAK method takes only 1.77 s. Consequently, SAK method can meet the time consumption requirement of the real-time Φ-OTDR system.

### 3.3. Discrimination of Perturbations

In Section 2.1, we analyzed the kurtosis of different types of signals in Φ-OTDR. The simulation results indicated that we may discriminate the random noise, instantaneous destructive perturbations, and stationary environmental interferences by kurtosis in simple scenarios. In this section, we performed two experiments to validate the simulation results. In addition to using pencil-break vibration and digging as the instantaneous destructive perturbations, we drove the PZT with various frequencies to simulate the interference signals.

**Figure 10:**
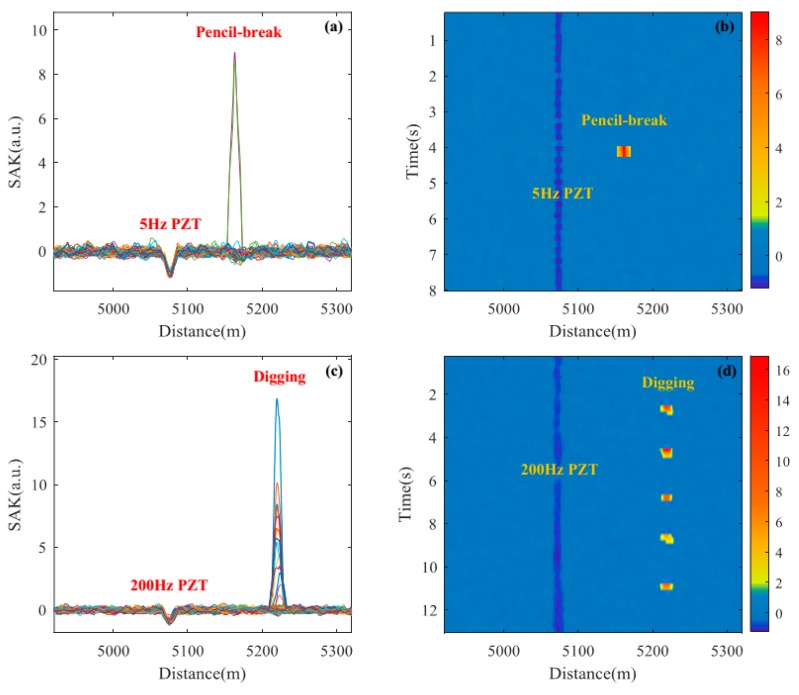
The location results of piezoelectric transducer (PZT) and instantaneous destructive perturbations (pencil-break and digging) through proposed method. (**a**) The SAK curves of 5 Hz PZT vibration plus pencil-break vibration; (**b**) The SAK image of 5 Hz PZT vibration plus pencil-break vibration; (**c**) The SAK curves of 200 Hz PZT vibration plus digging; (**d**) The SAK image of 200 Hz PZT vibration plus 5 digging events.

**Figure 11:**
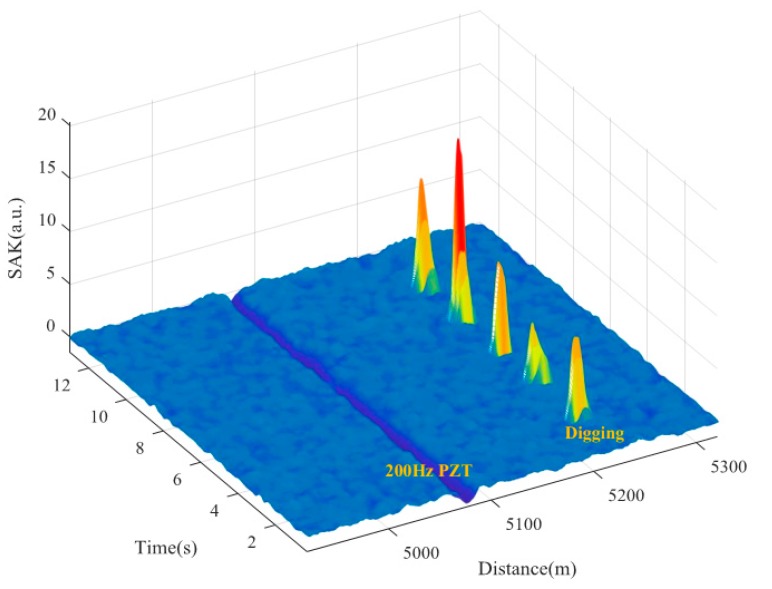
The three-dimensional SAK image of 200 Hz PZT vibration plus 5 digging events.

Figure 10 and Figure 11 show the locating results of various tests using SAK method. Figure 10a shows the SAK curves of 5 Hz PZT vibration plus pencil-break vibration. The SAK trough at ~5075 m and the SAK peak at ~5164 m locate the PZT vibration and the pencil-break vibration respectively. The SAK image of PZT plus pencil-break is shown in Figure 10b. One conspicuous dark stripe can be observed at ~5075 m, which indicates the PZT vibration. One bright point is observed at ~5164 m, which indicates the pencil-break vibration. Figure 10c,d show the locating results of 200 Hz PZT vibration plus five digging events. The SAK trough at ~5075 m and the SAK peak at ~5220 m in Figure 10c correspond to the PZT vibration and digging events respectively. The dark stripe at ~5075 m and 5 bright points at ~5220 m shown in Figure 10d, respectively, locate the PZT vibration and digging events as well, which can also be seen clearly in the three-dimensional SAK image (shown in Figure 11). It should be noted that when the PZT starts to work and stops working, the SAK may be high as well. Thus such “onset” and “offset” events could also be regarded as instantaneous destructive perturbations in that moment.

**Figure 12:**
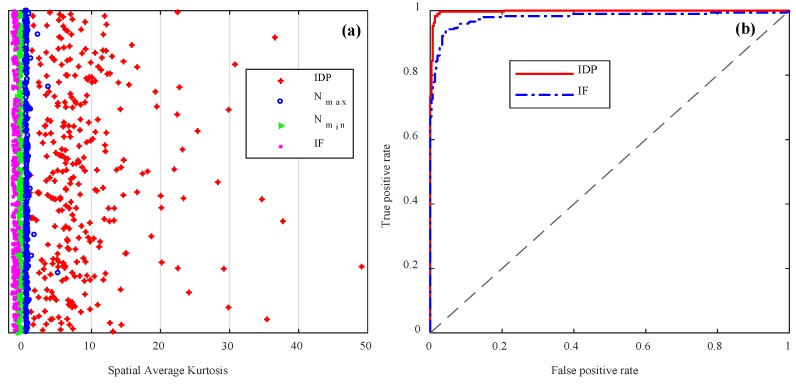
The statistic results of perturbation discrimination by SAK method. (**a**) The SAK distribution of different types of signals; (**b**) The receiver operating characteristic (ROC) curves of perturbation discrimination. IDP: instantaneous destructive perturbations; IF: interference.

In order to generally evaluate the discrimination performance of proposed method, we computed and analyzed the SAK value of a set of various tests (including 126 pencil-break vibrations, 168 digging events, 294 PZT vibrations with various frequencies, and 302 environmental noises). The statistic SAK result of various Φ-OTDR signals is shown in Figure 12a, where IDP means the instantaneous destructive perturbations (pencil-break vibration and digging event); N_max_ and N_min_ are the SAK maximum and SAK minimum of the noises; IF means interference (PZT vibration with different frequencies including 5, 50, 100 and 200 Hz). We can see that different types of signals have different SAK value interval.

We used true positive rate (TPR) and false positive rate (FPR) to evaluate the identification ability of the SAK method, where the TPR and FPR are defined as:

(8) $$\mathrm{TPR}=\frac{\mathrm{TP}}{\mathrm{TP}+\mathrm{FN}},\;\mathrm{FPR}=\frac{\mathrm{FP}}{\mathrm{TN}+\mathrm{FP}}.$$

Here, TP is the count of correct positive prediction, FP is the count of wrong positive prediction, TN is the count of correct negative prediction, and FN is the count of wrong negative prediction. The TPR is also known as the probability of detection, while the FPR is also known as the probability of false alarm. By plotting the TPR against FPR at various threshold settings, the receiver operating characteristic (ROC) curve is obtained, shown in Figure 12b. Four-fold cross-validation was used to evaluate the TPR and FPR of the perturbation discrimination by SAK. The SAK thresholds of discriminating the pencil-break (or digging) and PZT vibration were fitted on the training data of each fold using a logistic regression model. The evaluation results on test data of each fold are show in Table 3. 

We can see that the average TPR, FPR and accuracy of discriminating pencil-break or digging from noise are 95.57%, 1.02%, and 97.32%, respectively, while the average TPR, FPR, and accuracy of discriminating PZT vibration from noise are 92.54%, 4.38%, and 94.13%, respectively.

## 4. Discussion

Compared with the previous locating methods for Φ-OTDR systems, the SAK method has the following advantages:

(1) It can locate the instantaneous destructive perturbations with a higher SNR. This is because that the Φ-OTDR signals caused by instantaneous destructive perturbations generally have extreme value and result in large positive kurtosis, while noises in Φ-OTDR basically obey normal distribution and result in zero-kurtosis.
(2) It has better locating accuracy. By using a moving average on the spatial dimension, the SAK method can accurately locate the center of the vibration segment, even if the subsection is in an insensitive state, thus leading to more accurate locating results. It should be noted that “moving average on the spatial dimension” can be also used in other locating methods to fast figure out the accurate vibration segment center.
(3) It can simultaneously locate the perturbations and to some degree evaluate whether they are destructive or just interference. Because instantaneous destructive perturbations generally have higher positive kurtosis, while stationary interferences usually have lower negative kurtosis. The pencil-break, digging, and PZT discrimination results show that the SAK might be a promising feature for further event recognition.
(4) The time consumption of SAK method is short enough for a long distance real-time Φ-OTDR system. It should be noted that here the time consumption include both the locating time and discriminating time.

The SAK method has two main defects. One is that the localization time interval is directly related to the time step *s* in Equation (5). When the time step is 150 and the sampling rate is 1 kHz, the time interval of localization will be 0.15 s. However, for an Φ-OTDR threat alarm system this is totally acceptable. The other is that the SAK method only captures the short-time characteristics of the perturbations. Instantaneous destructive perturbations and stationary interferences might be discriminated by SAK, whereas long-term perturbations still require more features to determine whether they are a threat.

## 5. Conclusions

In this paper, we proposed a new perturbation localization and discrimination method for Φ-OTDR systems. Pencil-break vibration and digging events are used as the instantaneous destructive perturbations while PZT vibration is used to simulate interference. Experimental results show that, comparing with previous methods (MAD, TED, WD, MWD), the proposed method can locate the instantaneous destructive perturbations with a higher SNR. We also introduced moving average on the spatial dimension to improve the locating accuracy when coherent fading problem occur, thus leading to the smaller standard deviation of locating results. In addition, the SAK method can discriminate the instantaneous destructive perturbations from system noise and stationary environmental interference to some extent. Due to the little time consumption, SAK can be a promising real-time locating and perturbation discrimination method for Φ-OTDR systems.

## Figures and Tables

**Table 1:** The average locating SNRs of each method on the 200 locating tests.

Event	Pulse Width	MAD	TED	WD	MWD	SK	SAK
Pencil-break (dB)	100 ns	9.14	10.07	14.72	9.75	19.79	24.87
	500 ns	5.12	5.03	6.86	5.39	14.82	22.20
Digging (dB)	100 ns	8.70	11.57	14.31	11.18	16.22	22.04
	500 ns	4.56	6.42	8.93	6.12	15.72	23.81

**Table 2:** The average and standard deviation of the locating results under different pulse widths.

Event	Pulse Width	Statistics	MAD	TED	WD	MWD	SK	SAK
Pencil-break (m)	100 ns	Avg.	5163.9	5164.2	5164.3	5164.3	5163.6	5163.4
		Std.	1.7	1.9	1.7	1.6	2.3	**0.8**
	500 ns	Avg.	5165.8	5163.7	5164.6	5162.9	5162.4	5165.4
		Std.	9.6	8.6	9.9	8.7	10.3	**1.9**
Digging (m)	100 ns	Avg.	5219.7	5219.5	5219.3	5220.0	5218.5	5219.9
		Std.	1.3	1.2	1.1	2.0	2.1	**0.7**
	500 ns	Avg.	5220.4	5221.9	5221.3	5221.2	5219.0	5221.5
		Std.	11.6	11.0	10.9	10.3	11.6	**1.2**

**Table 3:** The true positive rate (TPR), false positive rate (FPR), and accuracy (Acc.) of signal discrimination using SAK method.

Events	Fold Index	TPR	FPR	Acc.
**Pencil-Break or Digging**	1	94.12%	1.23%	96.64%
	2	97.26%	1.32%	97.99%
	3	95.65%	0.00%	97.99%
	4	95.24%	1.54%	96.64%
	**Avg.**	**95.57%**	**1.02%**	**97.32%**
**PZT Vibration**	1	93.15%	6.58%	93.29%
	2	90.41%	1.32%	94.63%
	3	94.20%	2.50%	95.97%
	4	92.41%	7.14%	92.62%
	**Avg.**	**92.54%**	**4.38%**	**94.13%**
